# Nuclear Receptor SHP: A Critical Regulator of miRNA and lncRNA Expression and Function

**DOI:** 10.11131/2017/101312

**Published:** 2017-12-21

**Authors:** Yongfeng Song, Shan Lu, Jiajun Zhao, Li Wang

**Affiliations:** 1 Department of Endocrinology and Metabolism, Shandong Provincial Hospital affiliated to Shandong University, Jinan, Shandong, 250021, China; 2 Department of Physiology and Neurobiology, and the Institute for Systems Genomics, University of Connecticut, Storrs, CT 06269, USA; 3 Genesis Biotechnology, Trenton, NJ 08619, USA; 4 Veterans Affairs Connecticut Healthcare System, West Haven, CT 06516, USA; 5 Department of Internal Medicine, Section of Digestive Diseases, Yale University, New Haven, CT 06520, USA; 6 School of Pharmaceutical Sciences, Wenzhou Medical University, Wenzhou, Zhejiang 325035, China

**Keywords:** Nuclear receptors, Small heterodimer partner, non-coding RNAs

## Abstract

Small heterodimer partner (SHP, NR0B2) is identified as a unique orphan nuclear receptor that acts as a transcriptional repressor. SHP plays a crucial role in the control of various physiological processes and in several diseases by regulating the expression of disease-specific genes. Non-coding RNAs (ncRNAs), including long noncoding RNAs (lncRNAs) and microRNAs (miRNAs), are encoded of RNAs that are transcribed but not translated into proteins, which are involved in diverse developmental and cellular processes in eukaryotic organisms. Research during the past decade has identified factors participating in the regulation of ncRNAs biogenesis and function. In this review, we summarize recent findings demonstrating a critical role of SHP as a transcriptional regulator of ncRNAs expression and function.

## 1. Introduction

Small heterodimer partner (SHP, NR0B2) is a unique member of the nuclear receptor (NRs) superfamily [1]. It generally acts as a transcriptional repressor through interaction with other nuclear receptors or transcription factors (TFs) to inhibit function of these NRs or TFs on specific gene transcription, which contributes to its roles in diverse signaling pathways including metabolism and inflammation, and cell cycle [2]. Non-protein-coding RNAs (ncRNAs) account for more than 98% of the human genome, which play crucial roles in normal development and physiology [3]. NcRNAs are classified as long non-coding RNAs and small non-coding RNAs respectively based on the size of ncRNAs. Long non-coding RNAs are defined as ncRNAs that are longer than 200 nucleotides, while the length of small non-coding RNAs ranges from few to 200 nucleotides [4]. In the past, most studies were focused on elucidating the function and mechanism of ncRNAs, however, relatively little is known about the regulation of ncRNAs by NRs and TFs [5]. Growing evidence suggests that ncRNAs can be regulated at different levels, including promoter transcription, methylation, chromatin state regulation, and post-transcriptional regulation [6, 7]. In this mini-review, we will discuss the regulation of ncRNAs expression by SHP and to elaborate on their possible regulatory mechanisms.

## 2. The Basic Function of SHP

The most well established function of SHP in hepatic bile acid (BA) biosynthesis was demonstrated more than a decade ago using SHP knockout mice [8,9]. The increased BA levels in Shp-deficient mice could be in part associated with the increased energy metabolism and insulin sensitivity in other metabolic tissues including brown fat [10] and pancreas [11]. Interestingly, loss of SHP in leptin-deficient mice increased insulin sensitivity and diminished the severity of fatty liver [12], whereas overexpression of SHP in adipose tissue exacerbated high-fat diet-induced obesity [13]. In addition to inhibiting the rhythmic expression of BA synthetic enzymes Cyp7a1 and Cyp8b1 [14], SHP also directly represses the cholesterol biosynthesis enzyme, namely 3-hydroxy-3-methylglutaryl coenzyme A reductase [15]. On the other hand, SHP regulates hepatic glucose metabolism by disrupting AMPK-dependent repression of gluconeogenesis [16].

Liver fibrosis occurs due to the excessive accumulation of extracellular matrix proteins from activated hepatic stellate cells (HSCs) in response to liver injury. Loss of SHP sensitized liver to cholestatic liver fibrosis [17], which involved E2F1 and Egr1 transcription factors [18]. In terms of liver cancer, SHP expression was lost in human hepatocellular carcinoma (HCC) [19], which was likely attributed by SHP inhibition of hepatocyte proliferation [20], activation of apoptosis [21] and in the repression of epigenetic modifying enzymes [22, 23]. With regard to breast cancer, numerous studies provided the possibility that estrogen signaling would be specifically inhibited at multiple levels by SHP expression [24,25]. SHP as a tumor suppressor also interacts with other partners for its anti-tumor activity. Indeed, the transcriptional activity of glioma-associated oncogene homologue (Gli) was reported to be inhibited by SHP [26]. Recent studies showed that SHP is an essential negative regulator of the innate immune signaling. SHP repressed the inflammasome activation induced by toll-like receptor (TLR) [27] and NLR Family Pyrin Domain Containing 3 (NLRP3) [28] in macrophages. A small molecule activator of SHP showed strong effect in inhibiting HCC cell migration by suppressing chemokine (C-C motif) ligand 2 (Ccl2) [29]. Overall, SHP exerts a crucial function to protect liver against various insults and injury and is indispensable to maintain bile acid, lipid and glucose homeostasis.

## 3. SHP Regulation of ncRNAs

It is well established that ncRNAs exhibit a broad range of functions. However, less is known how ncRNAs gene transcription is controlled by NRs. Recent studies suggest that SHP plays a pivotal role in the regulation of ncRNA expression.

### 3.1. SHP in microRNA regulation

#### 3.1.1. miR-433 and miR-127

**Figure 1:**
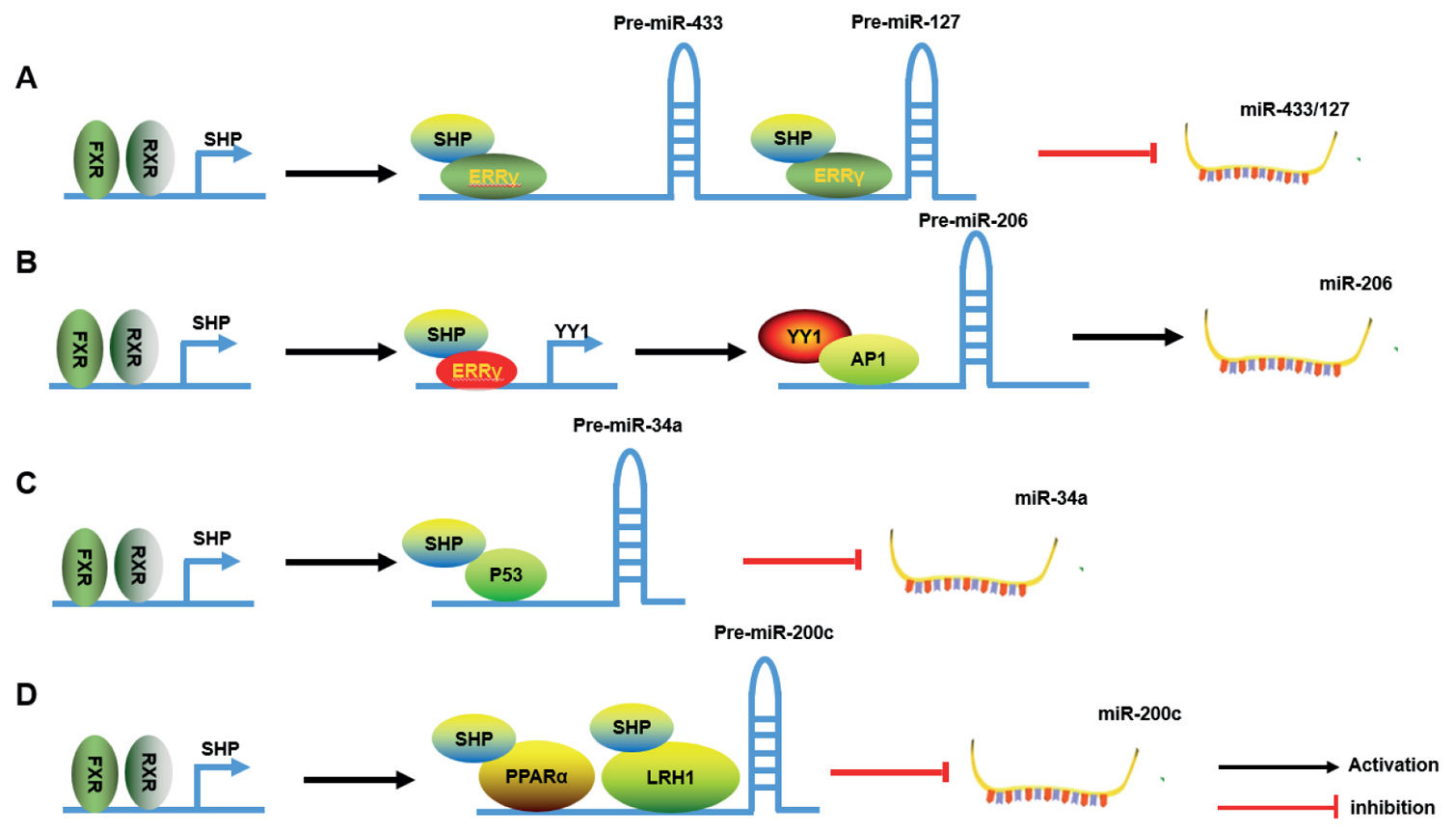
SHP as a transcriptional regulator of miRNA expression A) SHP inhibits ERRγ transactivation of the promoters of miR-433 and miR-127, which results in the repression of these two miRNAs. B) SHP activates miR-206 expression via a cascade dual inhibitory mechanism. The inhibition of ERRγ by SHP leads to decreased YY1 expression and the derepression of YY1 on AP1 activity, ultimately leading to the activation of miR-206. C) SHP inhibits p53 transactivation of the miR-34a promoter, resulting in the repression of miR-34a. D) SHP represses miR-200c expression by inhibiting the activity of PPARα and LRH-1.

miR-433 and miR-127 are part of a miRNA cluster but are expressed from independent overlapping primary transcripts [30]. The gene structures of the miR-433-127 loci are well conserved among multiple species [31]. Further study demonstrated that the miR-433 and miR-127 promoters were commonly activated by estrogen related receptor gamma (ERRγ) which was inhibited by SHP [32], providing the 1st evidence for the regulation of miRNA expression by SHP (Figure 1A). Interestingly, miR-433 inhibited liver cancer migration by targeting cAMP response element-binding protein (CREB) [33], whereas miR-127 inhibited HCC cell migration by targeting transforming growth factor-β (TGF-β)-mediated MMP13 [34]. A new study showed that miR-127 repressed high-mobility-group protein 2 (HMGB2) to modulate pluripotency of mouse embryonic stem cells and liver tumor initiating cells [35].

#### 3.1.2. miR-206

MiR-206, a member of the miR-1 family, was initially identified as a skeletal muscle specific miRNA [36] that played an important function in muscle development. For the regulation of miR-206 expression, TGF-β has been reported to inhibit miR-206 expression to regulate muscle differentiation [37]. We revealed a cascade “dual inhibitory” mechanism governing miR-206 gene transcription by SHP [38]. Specifically, ERRγ transactivated the promoter of YY1 (Ying Yang 1), which repressed the transcription factor AP1 (c-Jun and c-Fos)-induced miR-206 promoter activity. The SHP inhibition of ERRγ led to decreased YY1 expression and the derepression of YY1 on AP1 activity, ultimately leading to the activation of miR-206 (Figure 1B).

#### 3.1.3. miR-34a

Farnesoid X Receptor (FXR), the nuclear bile acid receptor, plays a pivotal role in maintaining bile acid homeostasis [39]. The miR-34a levels were elevated in FXR null mice but decreased in obese mice when FXR signaling was activated by FXR agonist GW4064 or FXR overexpression [40]. The expression of miR-34a was downregulated by FXR involving SHP. p53 is a key activator of miR-34a [41], which is destabilized by SHP [42] and Mdm2 [43]. When FXR is activated, SHP is recruited to the miR-34a promoter to inhibit p53 occupancy in the miR-34a promoter, thereby causing repression of miR-34a gene transcription in liver [40] (Figure 1C). Under normal conditions, miR-34a levels are down-regulated by the FXR/SHP cascade pathway. However, in the livers of obese mice, the FXR/SHP pathway is dysregulated and miR-34a levels are highly elevated, resulting in the reduced expression of its target gene SIRT1; an important regulator in the pathogenesis of metabolic disease [40].

#### 3.1.4. miR-200c

The miR-200c was initially identified to be distinctively expressed in the lung [44], which has been demonstrated to regulate key processes in tumorigenesis, including epithelial-mesenchymal transition (EMT), migration, invasion, stem cell maintenance, stromal remodeling and oxidative stress response [45]. Transcriptional regulation is the primary level of control for miR-200c expression. Zinc finger E-box-binding protein homeobox 1 (ZEB1), which was identified as a miR-200c target [46], was shown to repress miR-200c expression in a negative feedback loop [47]. The expression of miR-200c was activated by peroxisome proliferator activated receptor alpha (PPARα) and liver receptor homolog- 1 (LRH-1) but inhibited by SHP. Knockdown of SHP dramatically enhanced the ability of the LRH-1 agonist RJW100 to induce miR-200c. Furthermore, co-expression of PPARα and LRH-1 transactivated the miR-200c promoter, which was repressed by SHP co-expression, suggesting that SHP represses miR-200c expression by inhibiting the activity of PPARα and LRH-1 [48] (Figure1D).

### 3.2. SHP as a target of miRNAs

In spite of its critical role in the regulation of miRNA expression, SHP can also be a target of miRNAs. It was found that SHP was downregulated in multiple prostate cancer cell lines. The mature form of miR-141 was upregulated in prostate cancer cells. miR-141 can bind to SHP 3’ UTR resulting in translational suppression and RNA degradation, which modulates androgen receptor transcriptional activity [49].

## 4. SHP in LncRNA Regulation

Despite intensive efforts aimed at understanding the function of lncRNAs, little is known about how lncRNAs are regulated transcriptionally. Several recent studies shed lights on the role of SHP in lncRNAs expression regulation.

### 4.1. H19

H19 is imprinted and almost exclusively expressed from the maternally inherited allele [50]. The activation of H19 in various cancers including HCC and bladder carcinoma [51] has a significant influence on tumor growth. H19 expression is low in adult human liver but is highly induced in livers with cholestatic fibrosis and cirrhosis, indicating that H19 may be involved in the pathogenesis of liver fibrosis [52]. Hepatic overexpression of the anti-apoptotic protein Bcl2 caused SHP protein degradation. SHP inhibited *H19*RNA expression. Therefore, in Bcl2 over-expressed mice, H19 was markedly induced due to loss of SHP repression [52]. Further detailed studies showed that bile acid accumulation induced by bile duct ligation (BDL) increased hepatic *H19*RNA expression. The up-regulation of *H19*RNA enhanced immuno-cell infiltration, activated hepatic stellate cells, and promoted cholangiocyte proliferation, which facilitated the development of cholestatic liver fibrosis [53]. A most recent study identified a novel function of H19 in non-alcoholic fatty liver disease (NAFLD) by interaction with RNA binding protein polypyrimidine tract-binding protein 1 (PTBP1) to modulate hepatic lipogenesis and glucose metabolism [54]. In particular, *H19*RNA facilitated PTBP1’s association with SREBP1c mRNA and protein, leading to increased stability and nuclear transcriptional activity. Ectopic expression of H19 induced steatosis and pushed the liver into a “pseudo fed” state in response to fasting by promoting PTBP1-mediated SREBP1c protein cleavage and nuclear translocation. Deletion of H19 or knockdown of PTBP1 abolished high-fat and high-sucrose (HFHS) diet-induced steatosis.

### 4.2. MEG3

**Figure 2:**
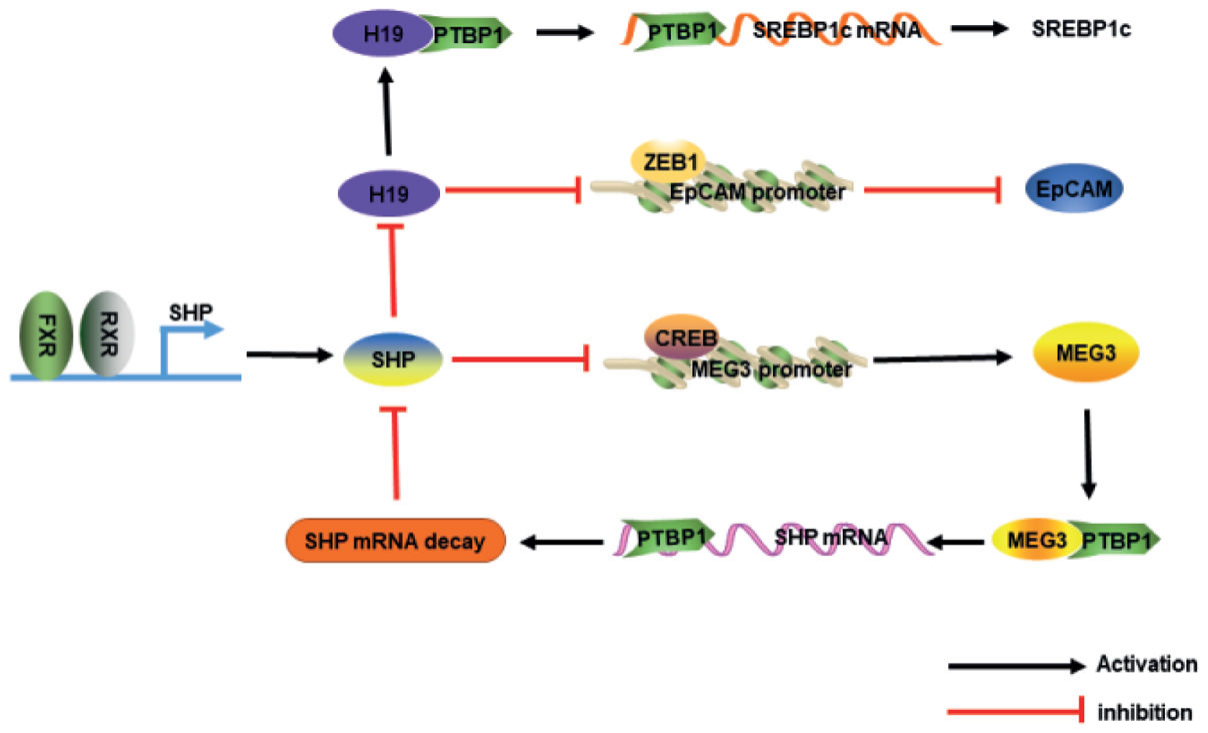
SHP as a transcriptional regulator of lncRNA expression SHP functions as a transcriptional repressor of both MEG3 and H19 expression. SHP represses CREB transactivation of the MEG3 promoter, resulting in the inhibition of MEG3 expression. In a feedback regulatory loop, MEG3 recruits PTBP1 to Shp mRNA, resulting in Shp mRNA decay. SHP also represses lncRNA H19 expression. H19 in turn inhibits ZEB1 binding to the EpCAM promoter, thus prevents the repressiive effect of ZEB1 on EpCAM transcription. H19 also recruits PTBP1 to Srebp1c mRNA to increase its stability, thus enhances lipogenesis.

Maternally expressed gene 3 (MEG3) is an imprinted gene and plays an important role in development and growth [55]. MEG3 RNA was dramatically elevated in the liver of *Shp^−/−^* mice compared with wile type mice, which was revealed by RNA-seq [56]. The MEG3 promoter was activated by ectopic expression of cAMP response element-binding protein (CREB), which was inhibited by SHP overexpression. Therefore, SHP inhibited MEG3 gene transcription by repressing transactivation of the MEG3 promoter [56, 57] (Figure 2). Interestingly, MEG3 interacted with RNA binding protein PTBP1 to cause SHP mRNA decay, thus providing a feedback mechanism to control SHP expression.

## 5. Conclusion

The diversity of ncRNAs shows a new level of the complexity of nature and makes ncRNA research relatively complex. However, due to the cell-specific expression pattern of ncRNAs, this new area gives us great opportunities to develop more personalized approaches for clinical applications and diagnosis. New lncRNAs related to human liver diseases are frequently identified which may serve as serum biomarkers [58]. More importantly, the pivotal role of SHP in the control of miRNAs and lncRNAs expression will enable new discoveries for future therapeutic intervention.
